# Predictive factors for relapse in triple-negative breast cancer patients without pathological complete response after neoadjuvant chemotherapy

**DOI:** 10.3389/fonc.2022.1016295

**Published:** 2022-12-01

**Authors:** Angela Toss, Marta Venturelli, Monica Civallero, Claudia Piombino, Federica Domati, Guido Ficarra, Francesca Combi, Eleonora Cabitza, Federica Caggia, Elena Barbieri, Monica Barbolini, Luca Moscetti, Claudia Omarini, Federico Piacentini, Giovanni Tazzioli, Massimo Dominici, Laura Cortesi

**Affiliations:** ^1^ Department of Oncology and Hematology, Azienda Ospedaliero-Universitaria di Modena, Modena, Italy; ^2^ Department of Medical and Surgical Sciences, University of Modena and Reggio Emilia, Modena, Italy; ^3^ Department of Surgery, Medicine, Dentistry and Morphological Sciences with Transplant Surgery, Oncology and Regenerative Medicine Relevance, University of Modena and Reggio Emilia, Modena, Italy; ^4^ Pathology Unit, University Hospital of Modena, Modena, Italy; ^5^ Unit of Breast Surgical Oncology, Azienda Ospedaliero-Universitaria di Modena, Modena, Italy; ^6^ Department of Biomedical, Metabolic and Neural Sciences, International Doctorate School in Clinical and Experimental Medicine, University of Modena and Reggio Emilia, Modena, Italy

**Keywords:** triple-negative breast cancer, neoadjuvant chemotherapy, pathologic response, residual cancer burden (RCB), multifocal disease

## Abstract

**Introduction:**

Triple-negative breast cancer (TNBC) patients who do not obtain pathological complete response (pCR) after neoadjuvant chemotherapy (NACT) present higher rate of relapse and worse overall survival. Risk factors for relapse in this subset of patients are poorly characterized. This study aimed to identify the predictive factors for relapse in TNBC patients without pCR after NACT.

**Methods:**

Women with TNBC treated with NACT from January 2008 to May 2020 at the Modena Cancer Center were included in the analysis. In patients without pCR, univariate and multivariable Cox analyses were used to determine factors predictive of relapse.

**Results:**

We identified 142 patients with a median follow-up of 55 months. After NACT, 62 patients obtained pCR (43.9%). Young age at diagnosis (<50 years) and high Ki-67 (20%) were signi!cantly associated with pCR. Lack of pCR after NACT resulted in worse 5-year event-free survival (EFS) and overall survival (OS). Factors independently predicting EFS in patients without pCR were the presence of multifocal disease [hazard ratio (HR), 3.77; 95% CI, 1.45–9.61; p=0.005] and residual cancer burden (RCB) III (HR, 3.04; 95% CI, 1.09–9.9; p=0.04). Neither germline BRCA status nor HER2-low expression were associated with relapse.

**Discussion:**

These data can be used to stratify patients and potentially guide treatment decision-making, identifying appropriate candidates for treatment intensi!cation especially in neo-/adjuvant setting.

## Introduction

Triple-negative breast cancer (TNBC) is defined by the lack of estrogen receptor (ER) and progesterone receptor (PR) expression and HER2 gene amplification. TNBCs present aggressive biology, with higher risk of local and distant recurrence compared to other subtypes, rapid progression with short response duration to therapies, and poor survival outcomes. They are typically diagnosed at younger age (1) and are more likely to present as a palpable mass becoming clinically apparent between annual screening mammograms (“interval cancer”) (2). At diagnosis, the majority of TNBC patients present with stage T2 or T3 and have involved lymph nodes and positive lymphovascular invasion (3). Metastatic diseases are more likely to occur in the viscera and brain compared to other breast cancer subtypes with a lower prevalence of bone metastasis (1). Moreover, most of the metastatic disease occurs within 2 or 3 years from diagnosis (1), whereas women who do not relapse during this time have similar survival rates of hormone receptor (HR)-positive BC.

Chemotherapy is the backbone for patients with TNBC in neoadjuvant (NACT), adjuvant, and metastatic setting, and despite its aggressive behavior, TNBC is particularly sensitive to cytotoxic chemotherapy (“triple negative paradox”). In the early stage, it is well established that the long-term outcome of neoadjuvant or adjuvant chemotherapy approach is the same. Nonetheless, NACT, initially used only to convert unresectable tumors into resectable ones, reduces the extent of surgery and improves cosmetic outcomes. Furthermore, the occurrence of a pathological complete response (pCR) after NACT emerged as an indicator of responsiveness to standard therapy. Indeed, patients who obtain pCR—defined as a lack of invasive disease in both breast and lymph nodes—showed improved outcomes in terms of event-free survival (EFS) and OS, whereas residual disease post-NACT is predictive of early recurrences and mortality (4, 5). For all these reasons, NACT became the standard of treatment also for patients with operable disease. However, most patients treated with standard anthracycline- and taxane-based NACT do not experience pCR (5, 6), and data are scarce regarding factors predictive of relapse in this subgroup of patients with residual disease after NACT. The present study aimed to identify factors predictive of relapse in patients with TNBC without pCR after NACT.

## Materials and methods

### Study population and design

A retrospective review of the electronic medical records of the Unit of Breast Surgery was performed, and 142 patients treated with NACT for early or locally advanced TNBC between January 2008 and May 2020 at the University Hospital of Modena were identified. All the patients with clinical data available, age ≥8 years, and diagnosed with early/locally advanced TNBC (T1–4, N0/+) who underwent NACT were included in the study. Exclusion criteria were diagnosis of hormone-receptor-positive and/or HER2-positive tumors, stage IV cancer, and primary surgery followed by adjuvant chemotherapy.

Tumor-specific characteristics, including tumor and nodal stage, histology, grade, and lymphovascular space invasion, were collected. Furthermore, patient, radiological, and treatment features were evaluated, including age at diagnosis and body mass index (BMI). In particular, data about breast MRI, cytohistological examination from fine-needle aspiration (FNA)/core biopsy, and multicentricity/multifocality or bilaterality were collected. Cytohistological examinations were determined by a pathologist at the time of surgery. Multicentricity/multifocality was defined as more than one foci of tumor in the same breast at the radiological examination before the surgery, independent of quadrant or distance.

The type of NACT, either within clinical trials or based on standard guidelines, and the type of breast and axillary surgery and adjuvant treatments were analyzed. Data about pathological downstaging and pCR, defined as no evidence of invasive disease in the breast and lymph nodes (ypT0/is, ypN0), were collected. In patients not achieving pCR, residual cancer burden (RCB) (7) and tumor-infiltrating lymphocytes (TILs) (8) were assessed. Time from NACT end to surgery and time from surgery to radiation therapy were included in this analysis. Finally, patients’ data concerning germline mutational status of *BRCA1* or *BRCA2* or about other breast/ovarian hereditary cancer syndrome genes, when available, were included as well.

This retrospective monocentric study was approved by our local Ethical Committee of Area Vasta Emilia Nord (Prot. AOU 25084/20).

### Statistical analysis and outcome measures

Baseline differences for clinical and demographic endpoints between patients with and without pCR were assessed by chi-square test or Fisher’s exact test (two-sided) for data analysis. A p-value < 0.05 was considered statistically significant. Outcomes of interest were event-free survival (EFS) and OS, and survival estimates were calculated and reported at 5 years. Time intervals were calculated from diagnosis until death or last follow-up. Patients were censored at the date of last clinical contact. EFS was defined as the time from the date of the diagnosis to the date of the first documented relapse (local, regional, and/or distant), while OS was defined as the time from diagnosis of BC to death/last follow-up. Overall survival and presence/absence of relapse was compared between patients with and without pCR after NACT using the Kaplan–Meier method. Survival estimates were calculated and reported at 5 years, along with their 95% confidence intervals (95% CIs), in the pCR and no pCR group. In patients without pCR, univariate and multivariable Cox analyses were used to determine factors predictive of relapse. Statistical analyses were done using IBM SPSS Statistics for Windows Version 23.0 (IBM Corporation, Armonk, NY, USA).

## Results

### Patients and treatment characteristics

A total of 142 patients were identified and included in this study. Median follow-up was 55 months (range, 7–155 months). The characteristics of TNBC patients are listed in **Table 1**. BMI at diagnosis was considered normal (range, 18.5–24.9) in 62.8% of patients. Of the patients, 73.2% underwent genetic testing, and 30% of them presented germline likely pathogenic or pathogenic variants in cancer predisposition genes (24 BRCA1, 4 BRCA2, 1 PALB2, 1 NBN, and 1 ATM). Most of the patients presented clinical tumor stage cT2 (75.2%) and no lymph nodes involvement (56%). TNBCs were predominantly monolateral (97.2%) and unifocal (71.8%).

**Table 1:** Characteristics of TNBC patients and histopathological tumor characteristics on tumor biopsy.

Characteristic	N(%)
Total	142
Age at diagnosis	
<50	68 (47.9%)
≥50	74 (52.1%)
* Unknown*	0
Genetic mutation	
No	73 (70.2%)
Yes	31 (29.8%)
* Unknown*	38
BMI	
<25	86 (62.8%)
≥25	51 (37.2%)
* Unknown*	5
Bilateral disease	
No	138 (97.2%)
Yes	4 (2.8%)
* Unknown*	0
Multifocal disease	
No	102 (71.8%)
Yes	40 (28.2%)
* Unknown*	0
Histology on tumor biopsy	
Ductal	131 (93.6%)
Other	9 (6.4%)
* Unknown*	2
Grade on tumor biopsy	
II	4 (3.3%)
III	119 (96.7%)
* Unknown*	19
Ki-67 on tumor biopsy	
<20	10 (7.1%)
≥20	132 (92.9%)
* Unknown*	0
HER2 on tumor biopsy	
0	57 (41.0%)
1+/2+(ISH negative)	82 (58.9%)
* Unknown*	3
Clinical T stage	
T1	19 (13.5%)
T2	106 (75.2%)
T3	6 (4.2%)
T4	10 (7.1%)
* Unknown*	1
Clinical N stage	
N0	80 (56.3%)
N+	62 (43.7%)
* Unknown*	0

Treatment characteristic, response to NACT, and histopathological tumor characteristic on the surgical tissue are shown in **Table 2**. A total of 109 patients (76.8%) underwent an anthracycline and taxane-based NACT, 6 patients (4.2%) underwent an anthracycline or taxane-based NACT, whereas 27 patients (19.0%) underwent platinum-based NACT. Overall, 60.6% had pre-treatment breast MRI. The average time between neoadjuvant chemotherapy and surgery was 31.4 days (range, 16–74). Seventy-four patients (53.6%) had mastectomy, 83 (58.9%) had sentinel lymph node biopsy (SLNB) performed, and 68 patients (48.6%) had at least 11 lymph nodes removed at the definitive surgery. pCR was obtained in 62 patients (43.9%). After NACT, most of the patients had high nuclear-grade residual (93.8%), high Ki-67 index (62.5%), and no lymphovascular space invasion (LVSI) (72.9%). In 72.9% of cases, Ki-67 index decreased after NACT.

**Table 2:** Treatment characteristics, response to neoadjuvant chemotherapy, and histopathological tumor characteristic on surgical tissue.

Characteristic	N (%)
Total	142
MRI	
No	56 (39.4%)
Yes	86 (60.6%)
* Unknown*	0
Time NACT-Surgery	Median 31.4 days (16–74)
<30 days	63
≥30 days	74
Type surgery	
Conservative	64 (46.4%)
Mastectomy	74 (53.6%)
* Unknown*	4
SLNB	
No	58 (41.1%)
Yes	83 (58.9%)
* Unknown*	1
Type of NACT	
Anthracycline and taxane-based	109 (76.8%)
Anthracycline or taxane-based	6 (4.2%)
Platinum-based	27 (19.0%)
* Unknown*	0
ypT	
0	67 (47.9%)
1	73 (52.1%)
* Unknown*	2
ypN	
0	117 (82.9%)
1	24 (17.1%)
* Unknown*	1
LN assessment	
≤10	72 (51.4%)
>10	68 (48.6%)
* Unknown*	2
pCR	
No	79 (56.1%)
Yes	62 (43.9%)
* Unknown*	1
Residual cancer burden	
I	3 (3.9%)
II	59 (77.6%)
III	14 (18.4%)
* Unknown*	3
TILs on residual tumor	
<30%	39 (51.3%)
≥30%	37 (48.7%)
* Unknown*	3
Grading on residual tumor	
II	4 (6.2%)
III	61 (93.8%)
* Unknown*	14
Ki-67 on residual tumor	
<20	27 (37.5%)
≥20	45 (62.5%)
* Unknown*	7
Ki67 pre- *vs*. post-NACT	
Stable/increased	19 (27.1%)
Decreased	51 (72.9%)
* Unknown*	9
LVSI on residual tumor	
No	35 (72.9%)
Yes	13 (27.1%)
* Unknown*	31
Adjuvant treatment	
No	112 (79.4%)
Yes	29 (20.6%)
* Unknown*	1
Time surgery-RT	Median, 84.6 days (43–171)
≤90 days	44
>90 days	21

MRI, magnetic resonance imaging; NACT, neoadjuvant chemotherapy; SLNB, sentinel lymph node biopsy; LN, lymph node; pCR, pathological complete response; LVSI, lymphovascular space invasion; RT, radiotherapy.

Adjuvant treatment was prescribed in 29 patients (20.6%) (capecitabine in 7 cases, immunotherapy in 6, capecitabine and immunotherapy in 1 case, olaparib in 3, and other chemotherapy agents in 11 patients), while adjuvant radiotherapy was performed in 86 patients (60.9%). The average time between surgery and radiotherapy was 84.6 days (range, 43–171).

### Outcomes

**Figure 1:**
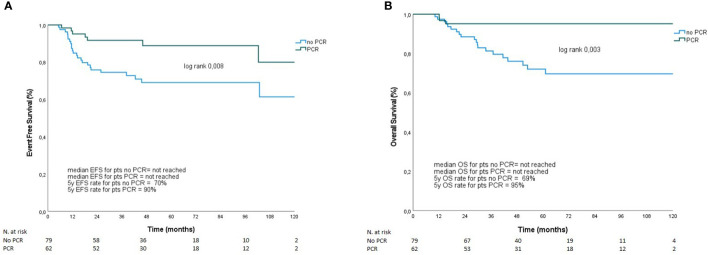
EFS **(A)** and OS **(B)** according to pCR. Overall survival and presence or absence of relapse was compared between patients with and without pCR after NACT using the Kaplan–Meier method. Survival estimates were calculated and reported at 5 years, along with their 95% confidence intervals (95% CI), in pCR and no pCR group. **(A)** Patients who obtained pCR after NACT showed 5-year EFS 90% *vs*. 70%, p=0.008. Panel **(B)** represents 5-year OS in patients with and without pCR (95% *vs*. 69%, p=0.003).

Seven out of 62 patients (9.7%) who achieved pCR relapsed (three patients with loco-regional disease and four with distant metastasis). Among the 80 patients who did not achieve pCR, one was lost to follow-up, and information regarding her tumor residual and outcome are is available. On the other hand, 23 out of 79 patients (29.1%) who did not achieve pCR relapsed. Of those, 12 patients had loco-regional relapse, and 11 had distant disease. As shown in **Figure 1**, patients who obtained pCR after NACT showed better EFS (5-year EFS 90% *vs*. 70%, p=0.008) and OS (5-year OS, 95% *vs*. 69%, p=0.003). Overall, young age at diagnosis (<50 years) and high Ki-67 (≥ 20%) were significantly associated with pCR (**Supplementary Table S1**). Moreover, although not statistically significant, pCR rate was higher with platinum-based NACT (51.8%) than without platinum agents (42.1%).

### Univariate and multivariable analyses

**Figure 2:**
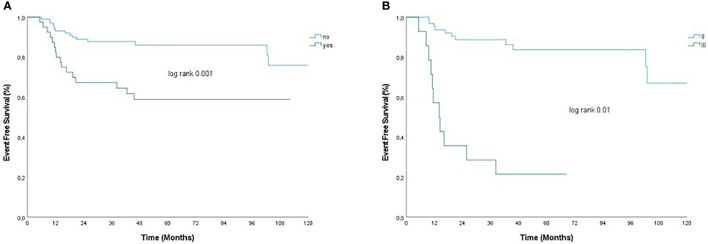
EFS by **(A)** multifocal disease and **(B)** RCB in patients without pCR after neoadjuvant chemotherapy. Panel **(A)** presents EFS for patients not achieving pCR with and without multifocal disease (yes/no) and panel **(B)** presents EFS for patients with RCB II *vs*. RCB III.

In univariate analysis, factors associated with relapse in the cohort of patients with residual disease after NACT (79 patients) were BMI > 25, bilateral BC, multifocal disease, clinical T3–T4 stage, clinical N+ stage, RCB III, LVSI, and prescription of adjuvant treatment (**Table 3**). Interestingly, although not statistically significant, there were trends that suggested an advantage of platinum-based NACT (hazard ratio, 0.05; 95% CI, 0.01–1.009; p=0.06) and worse outcome with TILs <30% (hazard ratio, 3.48; 95% CI, 0.96–12.5; p=0.06), and tumor grade III (hazard ratio, 4.2; 95% CI, 1.4–12; p=0.05) on tumor residual. Among significant factors, bilateral BC and LVSI were excluded from the multivariable analysis because of the few patients with bilateral disease and the rate of unknown data for LVSI. On multivariable analysis, multifocal disease (hazard ratio, 3.77; 95% CI, 1.45–9.61; p=0.005) and RCB III (hazard ratio, 3.04; 95% CI, 1.09–9.9; p=0.04) remained significant independent predictors of relapse. **Figure 2** presents EFS for patients not achieving pCR with and without multifocal disease and RCB II *vs*. RCB III.

**Table 3:** Univariate and multivariable analyses of EFS in patients not achieving pCR.

	*Univariate analysis*		*Multivariate analysis*	
*Characteristic*	*HR (95% CI)*	*P-values*	*HR (95%CI)*	*P-values*
Age at diagnosis				
<50	Ref			
≥50	0.7 (0.31–1.48)	0.34		
Genetic mutation				
No	Ref			
Yes	0.9 (0.6–1.6)	0.96		
BMI				
<25	Ref		Ref	
≥25	0.32 (0.12–0.8)	0.021	0.67 (0.23–1.94)	0.46
Bilateral disease				
No	Ref			
Yes	4.3 (1.3–14.5)	0.01		
Multifocal disease				
No	Ref		Ref	
Yes	3.2 (1.5–6.7)	0.002	3.77 (1.45–9.61)	0.005
MRI				
No	Ref			
Yes	0.7 (0.3–1.4)	0.36		
Histology on tumor biopsy				
Ductal	Ref			
Other	0.8 (0.19–3.4)	0.78		
Grade on tumor biopsy				
II	Ref			
III	1.001 (1–1.001)	0.19		
Ki-67 on tumor biopsy				
<20	Ref			
≥20	0.9 (0.99–1.006)	0.66		
Clinical T stage				
T1–T2	Ref		Ref	
T3–T4	3.4 (1.4–9)	0.02	1.35 (0.49–2.36)	0.07
HER2 low on tumor biopsy				
No	Ref			
Yes	0.7 (0.4–1.1)	0.16		
Clinical N stage				
N0	Ref		Ref	
N+	2.4 (1.1–5)	0.02	3.77 (1.48–9.63)	0.06
Time NACT surgery				
<30 days	Ref			
≥30 days	0.9 (0.94–1)	0.13		
Type surgery				
Conservative	Ref			
Mastectomy	1.8 (0.8–4.5)	0.14		
SLNB				
No	Ref			
Yes	1 (0.97–1.007)	1		
LN assessment				
≤10	Ref			
>10	1.01 (1.001–1.04)	0.05		
Type of NACT				
Anthra + tax	Ref			
Anthra/Tax	2.98 (0.87–10.1)	0.80		
Plat + other agents	0.41 (0.14–1.13)	0.85		
Anthra/tax	Ref			
Plat + other agents	0.05 (0.01–1.009)	0.06		
Residual cancer burden				
I	Ref			
II	21.8 (0.001–>100)	0.63		
III	0.58 (0.33–089)	0.04		
II	Ref		Ref	
III	8.57 (3.58–21.3)	0.001	3.04 (1.09–9.9)	0.04
TILs on residual tumor				
≥30	Ref			
<30	3.48 (0.96–12.5)	0.06		
Tumor grade on residual tumor				
II	Ref			
III	4.2 (1.4–12)	0.05		
Ki-67 on residual tumor				
<20	Ref			
≥20	0.61 (0.09–3.36)	0.81		
Ki-67 pre- *vs*. post-NACT				
Stable/increased	Ref			
Decreased	1 (0.99–1.01)	0.72		
LVSI on residual tumor				
No	Ref			
Yes	11 (3–36)	0.001		
Adjuvant CHT				
No	Ref		Ref	
Yes	2.9 (1.5–6)	0.03	1.89 (0.21–2.14)	0.54
Time surgery-RT				
≤90 days	Ref			
>90 days	0.99 (0.99–1)	0.09		

BMI, body mass index; MRI, magnetic resonance imaging; T, tumor; HER2, human epidermal growth factor receptor; N, node; NACT, neoadjuvant chemotherapy; SLNB, sentinel lymph node biopsy; LN, lymph node; Anthra, anthracycline-based chemotherapy; Tax, taxane-based chemotherapy; Plat, platinum-based chemotherapy; LVSI, lymphovascular space invasion; CHT, chemotherapy; RT, radiotherapy.

## Discussion

TNBC shows more aggressive features and has a poorer prognosis than other types of BC (1). In this subgroup of patients, the achievement of pCR after NACT represents one of the most important indicators of improved outcomes in terms of EFS and OS, whereas residual disease post-NACT is predictive of early recurrences and mortality (4, 5). Our study confirms the significant improved outcome (EFS and OS) of patients who obtained pCR after a standard NACT (mostly anthracycline, taxane, and/or platinum). Particularly, in our cohort, patients with pCR had a 5-year EFS of 90%, while those without pCR had a 5-year EFS of 70% [90% *vs*. 57% in the literature (9)]. On the other hand, the 5-year OS in patients with pCR was 95% compared to 69% of those without pCR (84% *vs*. 47% in the literature (9)). Furthermore, in line with previous literature (1), most of relapses in both our cohorts occurred within the first 5 years after the diagnosis.

The pCR rate after NACT in our study was 43.9%, which is consistent with data in the TNBC literature that typically range from 37% without platinum agents to 52.1% with the addition of platinum compounds (10). Although not statistically significant, also in our population, pCR rate was higher with platinum-based NACT (51.8%) than without platinum agents (42.1%), and this translated into a positive trend in EFS as well. Moreover, according to previous literature (11–14), younger age (<50 years) at diagnosis and high Ki-67 (≥20%) were associated with significantly increased pCR rate.

Up to 10%–20% of TNBC patients are found to carry deleterious germline BRCA1/2 mutations, and mutation prevalence is even higher in younger patients. Particularly, in a recent analysis of Italian TNBC patients diagnosed ≤60 years without breast and/or ovarian family history, BRCA detection rate was 22.6% (15). Although younger age (<50 years) at diagnosis predicts pCR in our analysis, germline mutational status does not significantly impact on pCR rate or on outcomes in the non-pCR cohort. According to the local testing criteria in use, 73.2% of patients included in this study underwent genetic testing, and 30% of them presented germline likely pathogenic or pathogenic variants in cancer predisposition genes (24 BRCA1, 4 BRCA2, 1 PALB2, 1 NBN, and 1 ATM). The high detection rate of pathogenic variants in this population is justified by the testing criteria that, before 2016, included only TNBC diagnosed before 40 years of age. On the other hand, the rate of pathogenic variants in the non-BRCA genes could be underestimated because multigene panel testing beyond BRCA genes was introduced in our institution only in 2018. Therefore, the evolution of testing criteria over the years may have influenced the detection rate of these gene mutations and may have an impact on the results of our analysis.

The univariable analysis showed a significant association between relapse and BMI > 25, bilateral BC, multifocal disease, clinical T3–T4 stage, clinical N+ stage, RCB III, LVSI, and prescription of adjuvant treatment. Moreover, although not statistically significant likely due to the small sample size, there were trends that also suggested worse outcome with TILs <30% and tumor grade III on tumor residual. With regard to presurgical characteristics, our results are in line with previous literature. Clinical stage at diagnosis was already shown to be one of the major risk factors for recurrence in breast cancer (16–18). Moreover, a recent meta-analysis suggested that overweight is associated with shorter disease-free and overall survival among TNBC patients (19). Among the residual disease features, our findings confirmed the prognostic role of LVSI and TILs in predicting relapse after NACT, as previously showed by an extensive body of literature (20–26). On the other hand, contrary to previous experiences (27–30), we did not observe an increased risk of relapse among patients with high post-treatment Ki-67 value. This discordance may be due to the great variability in Ki-67 evaluation, due to interlaboratory differences in staining methodology, scoring interpretation, and cutoff determination (31). As regards tumor grade, most of the previous studies evaluated tumor grade on biopsies at diagnosis and reported discordant results (32, 33). We did not find a significant association between pretreatment tumor grade and relapse, but a negative trend has been observed in grade III on tumor residual that should be further evaluated in larger cohorts.

Interestingly, in a multivariate model (including BMI > 25, multifocal disease, clinical T3–T4 stage, clinical N+ stage, RCB III, and prescription of adjuvant treatment), BMI, clinical stage, and adjuvant treatment lost significance. The multivariable analysis showed that, in the cohort of patients not achieving pCR, multifocal disease was associated with relapse. For purposes of this analysis, multifocality was defined as the presence of more than one foci in the same breast, regardless of whether they were in the same quadrant or of the distance between the lesions. The role of multifocality in breast cancer is still controversial, and some groups reported a higher rate of relapse and worse outcomes in multifocal tumors, whereas other groups showed that multifocality is not an independent predictor of prognosis in multivariate analysis (33–36).

The multivariable analysis also revealed that RCB III was associated with higher risk of relapse in patients with residual disease after NACT. The RCB score uses the diameter of residual disease, percentage of vital tumor cells, and diameter of the largest involved lymph node to calculate the amount of residual disease. This score has been validated with three distinct prognostic RCB classes in all BC subtypes, with the most significant discriminatory power in TNBC and HER2-positive BC (7, 37, 38). Indeed, our findings are consistent with previous literature and suggest that prospective evaluation of RCB could be considered to become part of standard pathology reporting after NACT, as also recently recommended by the International Collaboration on Cancer Reporting (39). The binary outcome of pCR versus residual disease confers little information, offering no distinction among patients with varied amounts of residual disease. The RCB score has the potential to be used in predicting a patient’s residual risk after NACT in a prospective setting, especially given the increasing options for adjuvant therapy in the setting of residual disease.

Age at diagnosis, germline predisposing gene mutations, BMI, breast MRI, histological subtype, grading, Ki-67, HER2 expression, clinical T or N stage, type of breast or axillary surgery, type of NACT, TILs in the residual tumor, time from NACT end and surgery, and time from surgery to radiation therapy and adjuvant therapy were not independent predictors of relapse in our cohort. Particularly, post-NACT adjuvant systemic treatment did not significantly impact on relapse in our multivariable analyses. In the last decade, several therapies have been investigated as adjuvant strategies in TNBC patients not achieving pCR, including capecitabine and olaparib (40–42). Additionally, immunotherapy is under evaluation in this setting in several clinical trials. Our findings suggest that, despite the addition of further treatments after NACT, the EFS of patient not achieving pCR remains poor, and no improvement has been obtained with the introduction of further adjuvant therapies. According to our findings, the achievement of pCR should remain as the primary aim in these patients and should be pursued by optimizing NACT, for instance by the addition of platinum agents (10, 43) or pembrolizumab (44).

Our study presents some limitations that should be highlighted. First, as a retrospective study, our analysis was limited by selection bias. The sample size is small and derives from a single institution; therefore, our patient population may not accurately represent the patterns of care at other institutions. Additionally, patients received a range of neoadjuvant therapies, and we did not control for the duration of treatment or the delay of dose in this analysis. However, a previous analysis of the I­SPY 2 trial suggested that the prognostic association of both pCR and RCB score is strong, regardless of the type of chemotherapy­based treatment (45). Finally, some data for each variable were missing in our medical records.

To conclude, our study confirmed the poor prognosis of TNBC patients who do not experience pCR after NACT. The challenge nowadays is to define the treatment paradigm for most of the patients who do not obtain pCR. Waiting for more accurate molecular characterization, multifocality, and RCB remain the most significant risk factors independently predicting relapse among patients without pCR. These data can be used to stratify patients in our clinical practice, potentially guiding treatment decisions and intensifying neo-/adjuvant treatments in patients at higher risk of relapse. Prospective clinical trials are needed to explore novel therapeutic approaches aimed at increasing the rate of pCR and improving adjuvant strategies for this high-risk cohort of patients.

## Data availability statement

The data analyzed in this study is subject to the following licenses/restrictions: Dataset is available upon reasonable request. Requests to access these datasets should be directed to angela.toss@unimore.it.

## Ethics statement

The studies involving human participants were reviewed and approved by Ethical Committee of Area Vasta Emilia Nord. The patients/participants provided their written informed consent to participate in this study.

## Author contributions

MV, CP, EC, FD, EB, CO, MB, LM, FCa and FCo provided clinical data. MV and AT drafted the manuscript. MC performed the statistical analysis. MV, AT, LC and GT conceived the study and interpreted the results. MV, AT, LC, FP and MD revised the manuscript. All authors contributed to the article and approved the submitted version.

## Acknowledgments

The authors especially thank the “Angela Serra Association for Cancer Research” for the support in this study.

## Conflict of interest

The authors declare that the research was conducted in the absence of any commercial or financial relationships that could be construed as a potential conflict of interest.

The reviewer EP declared a past co-editorship with one of the authors AT to the handling Editor.

## Publisher’s note

All claims expressed in this article are solely those of the authors and do not necessarily represent those of their affiliated organizations, or those of the publisher, the editors and the reviewers. Any product that may be evaluated in this article, or claim that may be made by its manufacturer, is not guaranteed or endorsed by the publisher.
