# Supplementary material for: Predictive factors for relapse in triple-negative breast cancer patients without pathological complete response after neoadjuvant chemotherapy
Source: Front Oncol. 2022 Dec 1;12:1016295. doi: 10.3389/fonc.2022.1016295 (PMC9753128; doi:10.3389/fonc.2022.1016295)
Supplement: Supplementary file 1 [file Table_1.docx]

| ***Characteristic*** | ***PCR*** | ***No PCR*** | ***P values*** |
| --- | --- | --- | --- |
| **Total** | **62** | **79** |  |
| Age at diagnosis  <50  >50  *unknown* | 48 (30-78)  39 (62.9%)  23 (37.1%)  0 | 53 (25-80)  28 (35.4%)  51 (64.6%)  0 | <.05 |
| Genetic mutation  No  Yes  *unknown* | 31 (60.8%)  20 (39.2%)  11 | 41 (78.8%)  11 (21.2%)  27 | >.05 |
| BMI  <25  >25  *unknown* | 37 (63.8%)  21 (36.2%)  4 | 48 (61.5%)  30 (38.5%)  1 unknown | >.05 |
| Bilateral disease  No  Yes  *unknown* | 61 (98.4%)  1 (1.6%)  0 | 76 (96.2%)  3 (3.8%)  0 | >.05 |
| Multifocal disease  No  Yes  *unknown* | 49 (79.1%)  13 (20.9%)  0 | 52 (65.8%)  27 (34.2%)  0 | >.05 |
| RMN  No  Yes  *unknown* | 20 (32%)  42 (68%)  0 | 35 (44.3%)  44 (55.7%)  0 | >.05 |
| Histology  Ductal  Other  *unknown* | 60 (96.8%)  2 (3.2%)  0 | 70 (90.9%)  7 (9.1%)  2 | >.05 |
| Grade  II  III  *unknown* | 1 (1.7%)  58 (98.3%)  3 | 3 (4.7%)  61 (95.3%)  15 | >.05 |
| Ki67  <20  >20  *unknown* | 1 (1.6%)  61 (98.4%)  0 | 9 (11.5%)  69 (88.5%)  1 | <.05 |
| HER2  0  1+/2+ (ISH negative)  *unknown* | 25 (41.7%)  35 (58.3%)  2 | 31 (39.7%)  47 (60.3%)  1 | >.05 |
| Clinical T stage  T1-T2  T3-T4  *unknown* | 56 (91.8%)  5 (8.2%)  1 | 68 (86.1%)  11 (13.9%)  0 | >.05 |
| Clinical N stage  N0  N+  *unknown* | 32 (51.6%)  30 (48.4%)  0 | 48 (60.8%)  31 (39.2%)  0 | >.05 |
| Time NACT_Surgery | Median 33 days (18-74)  < 30 days = 26  > 30 days = 34 | Median 33 months (16-56)  < 30 days = 37  > 30 days= 40 | >.05 |
